# The Impact of Drugs and Substance Abuse on Viral Pathogenesis—A South African Perspective

**DOI:** 10.3390/v16060971

**Published:** 2024-06-17

**Authors:** Lufuno Ratshisusu, Omphile E. Simani, Jason T. Blackard, Selokela G. Selabe

**Affiliations:** 1 HIV and Hepatitis Research Unit, Department of Virology, Sefako Makgatho Health Sciences University, Pretoria 0208, South Africa; omphile.simani@smu.ac.za (O.E.S.); blackajt@ucmail.uc.edu (J.T.B.); gloria.selabe@smu.ac.za (S.G.S.); 2 Division of Digestive Diseases, Department of Internal Medicine, University of Cincinnati College of Medicine, Cincinnati, OH 45267-0595, USA; 3 National Health Laboratory Service, Sefako Makgatho Health Sciences University, Pretoria 0208, South Africa

**Keywords:** South Africa, illicit drug, HIV, HBV, HCV, immune system

## Abstract

Illicit drug and alcohol abuse have significant negative consequences for individuals who inject drugs/use drugs (PWID/UDs), including decreased immune system function and increased viral pathogenesis. PWID/UDs are at high risk of contracting or transmitting viral illnesses such as human immunodeficiency virus (HIV), hepatitis B virus (HBV), and hepatitis C virus (HCV). In South Africa, a dangerous drug-taking method known as “Bluetoothing” has emerged among nyaope users, whereby the users of this drug, after injecting, withdraw blood from their veins and then reinject it into another user. Hence, the transmission of blood-borne viruses (BBVs) is exacerbated by this “Bluetooth” practice among nyaope users. Moreover, several substances of abuse promote HIV, HBV, and HCV replication. With a specific focus on the nyaope drug, viral replication, and transmission, we address the important influence of abused addictive substances and polysubstance use in this review.

## 1. Illicit Drugs of Abuse and Viral Infections

Globally, the use of illicit drugs has a significant negative impact on society, resulting in years of premature death or disability [1]. Substance use disorder (SUD) can be attributed to social pressure, peer pressure, dysfunctional families, genetic factors, emotional problems, mental health problems, loss of respect, trauma experience, loss of livelihood, and prior substance exposure [2,3]. People who use drugs (PWUDs) have high mortality rates compared to the general population globally [4,5]. SUD continues to be a major obstacle in combating the global pandemic of viral infections. According to the National Institute on Drug Abuse, PWUDs who engage in risky drug-related behaviours experience elevated risk of becoming infected with or transmitting viral infections such as human immunodeficiency virus (HIV) and viral hepatitis [hepatitis B virus (HBV), and hepatitis C virus (HCV)] [6]. There are two primary ways in which these viral infections can be acquired in this population: (1) when people inject drugs and share contaminated needles or other drug equipment, and (2) when drugs impair judgment and people engage in unprotected sexual activities with an infected partner [6]. Drug use and addiction have been associated with HIV/acquired immune deficiency syndrome (AIDS) since its first recognition. According to the Centers for Disease Control and Prevention (CDC), injecting drug use contributed to almost 20% of documented male HIV cases and 21% of female HIV cases in 2016 [7]. A global meta-analysis reported that 18% of people who inject drugs (PWIDs) were living with HIV, 52% were HCV-seropositive, and 9% were HBV surface antigen (HBsAg)-positive [8]. Table 1 shows a summary of studies conducted worldwide on drugs of abuse, such as prescription opioids, heroin, methamphetamine or amphetamine-type stimulants (ATSs), cocaine, crack cocaine, tranquilizers, stimulants, sedatives, tramadol, and pethidine, and their association with high-risk exposure to blood-borne viruses (BBVs) in PWIDs [9,10,11,12,13,14,15]. PWIDs experience a significant burden of BBVs compared to the general population. The aim of this review is to (1) highlight what is known about the prevalence of BBVs in SUD and drug abuse, and to (2) address the significant impact of abused addictive drugs and polysubstance use on viral replication and transmission with a specific focus on nyaope—a novel psychoactive drug that is on the rise in South African settings.

## 2. Substance Abuse in Africa

African countries experience different levels of drug abuse, with some regions experiencing higher rates than others. Multiple factors contribute to drug abuse, including poverty, unemployment, social inequality, and lack of access to education and healthcare [3,16]. The abuse of illicit drugs such as cocaine, methamphetamine, and heroin is a major problem in many urban areas worldwide, including South Africa [17]. Therefore, in many parts of the world, substance abuse among young people remains a significant public health concern. Commonly abused substances include alcohol, cocaine, heroin, marijuana (cannabis), tobacco, methamphetamine, and opioids (see Table 1). Recent studies conducted in Africa demonstrate that substance abuse is highly prevalent among young people, which results in physical and psychological problems such as fighting, vandalism, theft, engaging in unprotected sex, medical problems, personal injury, and impaired relationships [18,19,20]. Moreover, using more than one substance is common among substance users. In Africa, illicit substances such as cannabis (the most commonly used substance, present in West and Central Africa between 5.2% and 13.5%), amphetamine stimulants, and benzodiazepines are on the rise [21]. Additionally, alcohol, tobacco, cannabis, heroin, and Khat (*Catha edulis*) are the most common drugs abused by young people in sub-Saharan Africa [22,23]. As a result, Africa has been recognised in the last decade as a consumer and destination for illicit drugs [24,25]. A systematic review reported the overall prevalence of any substance use among adolescents in sub-Saharan Africa to be 42% and 56% in Central Africa [23]. Alcohol and tobacco are the most prevalent substances across the continent at 41% and 46%, respectively [23]. The use of illicit drugs is associated with a high risk of contracting or transmitting viral illnesses such as HIV, HBV, and HCV among PWID/UDs

## 3. Substance Abuse and Associated BBVs in South Africa

South Africa has the highest rate of drug consumption in Africa and is nearly twice that of the rest of the world [26]. According to the South African national estimates, 10% of the adult population (ages 15 years and older) consume harmful amounts of alcohol (17% of men and 5% of women) and 9% (13% of men and 4% of women) use illicit drugs [27]. In a study conducted in five cities across four South African provinces [Gauteng (from sites in Centurion, Pretoria, and Johannesburg), KwaZulu-Natal (from sites in Durban), and Western Cape Province (from sites in Cape Town)], 14% of PWIDs were infected with HIV in 2013 [9]. However, there is limited research on HBV and HCV among PWIDs in South Africa (Table 1). Most South African studies about PWIDs are limited to programmatic data and small, single-site studies. A study conducted in Cape Town reported an HCV seroprevalence of 27% among men who have sex with men (MSM) who use drugs, of which 80% injected heroin or methamphetamine in the previous 3 months [11]. Although there are limited data, the number of PWIDs in South Africa exceeds 75,000, and this key population accounts for ~1% of new HIV infections according to the South African National AIDS Council’s mid-term report for 2019 [28]. According to reports by Scheibe et al., Tshwane has the highest HCV prevalence (55%) among PWIDs, the highest HIV prevalence (21%), and a 5% HBV prevalence, primarily from homeless black men from the three major South African cities [10]. Therefore, it is not surprising that an increase in viral infections is associated with intravenous drug use as observed in other studies worldwide [12,13,14,15,29,30,31,32]. In South Africa, substance abuse is a serious and complex problem, with varying substances being abused, such as illicit street drug cocktails known as nyaope.

## 4. Nyaope—A Highly Addictive Novel Psychoactive Drug in South Africa

Nyaope is the street name that is commonly used in South African settings to describe a heroin-based powder mixture that is mostly commonly smoked with cannabis [33,34,35,36,37]. Media reports suggest that nyaope has been used as early as 2000 [38], although there is a lack of empirical evidence to support or refute this timeline. Instead, from 2006 and later, papers were published that provided concrete evidence of recreational nyaope use [17,39,40,41]. In South Africa, nyaope has emerged as a new illicit drug that is highly addictive, very potent, and commonly abused by young people and mostly from poor backgrounds, predominately from black townships (ranging between the ages of 15 and 64 years) [34,42,43]. Fifteen percent of South African youth engage in drug use, and the chances of encountering nyaope are higher [44]. In response to the country’s growing use of nyaope, the South African government criminalised the possession and distribution of nyaope in 2014 [36]. In addition, the sale of nyaope carries a potential 25-year prison term [36].

Nyaope is almost exclusively found in South Africa. It is most commonly used by young and unemployed black people from poor socioeconomic backgrounds due to its low cost (±ZAR 30 as of April 2018 per joint), and is the most common form of heroin used among black South Africans [34]. Nyaope is highly addictive, and once one becomes dependent on the drug, it is difficult to quit [34]. This has been shown by users who suffer from severe withdrawal symptoms (severe unbearable cramps) when they try to quit on their own [17,45,46]. Nyaope is an extremely potent narcotic drug compared to other well-known drugs; nevertheless, the chemical composition of nyaope has changed over time [47]. It frequently contains compounds including antiretrovirals (ARVs), cannabis, heroin, rat poison, and detergent [48,49,50]. Recreational drug mixtures containing ARVs may also contain harmful substances such as rat poison, household cleaning supplies, milk powder, pool cleaner, and bicarbonate of soda [51,52]. Chemical profiling of the compounds present in nyaope mixtures is limited to a small number of studies [17,38,39,40,41,49,52,53,54,55] (see Table 2). This drug cocktail is commonly referred to as nyaope [17,34,35,36,39,41,43,44,46,47,52,54,56,57,58,59,60,61,62,63,64,65,66,67,68,69,70,71,72]. According to prior media reports and studies, this drug cocktail has also been referred to by other names, including whoonga [38,39,41,46,49,52,54,59,62,63,64,68,70,71,73], wunga [46,62,64], plazana [53], kataza [40,52,68], unga [17,53,70], BoMkon [41,53], and sugars or pinch [35,68,70]. Whoonga, also known as wunga in isiZulu, was the subject of multiple news stories in 2010 [74]. According to many reports, it was purported to be a combination of illegal substances like methamphetamine, heroin, and/or marijuana, as well as ARVs (efavirenz) [75,76]. All of these names refer to cocktails containing heroin and cannabis in some form and are introduced to disguise the illegal drugs from law enforcement. The complete characteristics of the studies that have studied the impact of nyaope are summarised in Table 2.

## 5. The Transition of Using Nyaope from Smoking to Injecting Nyaope

Previously, nyaope concoctions have been known to be smoked with cannabis; however, recent reports now suggest the injection of nyaope either intravenously and via subcutaneous administration (e.g., dissolving nyaope powder in water and injecting it directly into the user’s veins) [33,37,43,56]. Nyaope users are commonly exposed to blood through the sharing of blood products via illicit and unsafe contaminated injections, needles, and syringes. In South Africa, this pattern of practice is known as “Bluetooth”, and it entails sharing of drugs by injecting a syringe full of blood directly into another person immediately after the initial injection. This pattern has also been identified in HIV-positive injecting drug users (IDUs) in Tanzania, where it is known as “Flashblood” [78,79]. According to media reports, “Bluetooth” is a new habit that has recently developed among IDUs which is a very dangerous practice of sharing blood that exacerbates the transmission of blood-borne viruses (BBVs) [80,81]. It is believed that the process of transitioning from smoking to injecting is a way to cope with a variety of resource constraints, as well as to satisfy curiosity and pleasure [82]. According to the media report, the “Bluetooth” sharing of drugs occurs when nyaope addicts and their peers do not have money to buy the drug and tend to share blood from an already drugged user as a way to feed each other’s cravings [83,84]. Therefore, the drug powder will be first mixed with water and drawn into a syringe. Thereafter, the first person will inject the drug into his veins and immediately after injection withdraw the blood and share it with the next person [83,84]. According to users, injecting the drug is more potent than smoking it [83,84]. Currently, injecting nyaope is on the rise in most regions of South Africa [73]. Other studies about drugs of abuse and their association with BBVs besides nyaope have been conducted worldwide (see Table 1). A study conducted in the United States of America (USA) in patients with opioid use disorder and injected drugs found that these patients are at an increased risk of HIV and HCV infections compared to the general population, with prevalence rates of 53% vs. 3% and 6% vs. 2%, respectively [13]. Another study by Cai and colleagues reported 11% HIV, 78% HBV, and 71% HCV infections among people who reported having used methamphetamine, cocaine, or heroin at some point in their lives [14]. To date, South Africa has limited data available on the dangers of using nyaope and its association with BBVs. Therefore, the abuse of illicit drugs via injection has a high risk of exposure to BBVs in PWIDs. 

## 6. Risk Factors Associated with Using Nyaope

BBVs such as HIV, HBV, and HCV are the most common viral infections in PWIDs because of the common route of transmission [10,82]. Hence, high-risk practices of sharing contaminated needles, syringes, and other drug-injection equipment put PWIDs at a high risk of acquiring and transmitting HIV, HBV, and HCV infections [10,13]. In addition, nyaope use in South Africa is growing at an alarming rate and becoming a public health concern. Most data about nyaope abuse are derived from public media reports. According to the media publication, approximately 45% of individuals who abuse nyaope via “Bluetooth” are HIV-infected [80]. In another recent study of individuals who practiced the “Bluetooth” method of sharing drugs, 35% were HIV-positive [81]. Thus, the “Bluetooth” sharing of nyaope exacerbates the transmission of viral infections. 

A wide range of clinical manifestations has been observed in nyaope users, including diarrhoea, facial swelling, vomiting, stomach cramps, erectile dysfunction, vein damage, right heart failure, and cortical atrophy [46,47,58,70]. Recently, two studies included nyaope users who used the drug cocktail while pregnant in South Africa (see Table 2) [59,65]. Among pregnant nyaope users, one infant died, whose cause of death was associated with the mother’s nyaope usage [59]. Other infants born to mothers who used nyaope suffered from septic shock, respiratory distress, seizures, multi-organ dysfunction, and retinopathy of prematurity [59,65].

## 7. Drugs of Abuse and the Immune System

Unregulated/illicit drugs of abuse impair immune system functions, causing the immune system to be more vulnerable to infectious diseases when illicit drugs are added to cells of the immune system in vitro [85,86]. There is growing evidence that drugs such as methamphetamine, cocaine, opioids, and marijuana increase HIV, HBV, and HCV replication both in vitro and in animal models [87,88,89]. Several mechanisms contribute to the decreased ability to control pathogens and limit their subsequent clearance, including the impaired function of natural killer cells, T-cells, B-cells, neutrophils, dendritic cells, macrophages, the altered expression of cytokines and chemokines, as well as weak intestinal barrier integrity [86,90]. Drugs of abuse can impair immune system functions, affecting both innate and adaptive immunity. An array of mechanisms contributes to the immunomodulatory effects of drugs of abuse, including direct cellular toxicity, the dysregulation of immune signalling pathways, and alterations in inflammatory responses, as reviewed by [86,90]. 

## 8. Impact of HIV on Cells of the Immune System

In vitro studies from various laboratories have also provided direct evidence that opiates (such as morphine) promote the HIV infection of HIV target cells [87,88,89]. Morphine has been shown to stimulate HIV replication within human monocytes/macrophages [91,92,93], T lymphocytes [94,95], Kupffer cells [96], human neuroblastoma cells [97], and human brain cells [98,99]. Dr. Blackard’s research group has also shown that the synthetic opioid fentanyl enhances HIV replication in several cell types through the enhanced expression of the CCR5 and CXCR4 chemokine receptors [88,89]. Therefore, these studies are paving the way for a better understanding of drug use and HIV/AIDS comorbidities. However, there are no data regarding the impact of nyaope on viral pathogenesis to date.

## 9. Drugs of Abuse and HIV Infection

Globally, South Africa carries the highest prevalence of HIV infection, and this is due to a variety of biological, socio-behavioural, contextual, and structural factors [100,101,102,103]. Increasing access to HIV treatment has been the country’s priority in recent years. As a result of antiretroviral therapy (ART), mortality and morbidity have decreased, and lifespans have increased among people living with HIV. Adherence to HIV treatment is an important determinant for achieving the suppression of the virus and preventing drug-resistant strains from occurring. However, adherence to the daily intake of HIV treatment remains a challenge [104]. The South African National Department of Health implemented an effective programme in 2010 to provide free ARVs to HIV-infected South Africans [105]. In 2014, The Joint United Nations Programme on HIV/AIDS (UNAIDS) launched the 95-95-95 goals. The goal was to diagnose 95% of all HIV-positive individuals, provide ART to 95% of those diagnosed, and achieve viral suppression for 95% of those treated by 2030 [106]. Because of ARVs being widely available, South Africa has been experiencing a rise in the recreational use of ARVs.

The recreational use of ARVs other than for the treatment of HIV infection has raised concerns, and understanding this phenomenon requires an examination of the physiological effects of these drugs. While not all ARVs have neuropsychiatric effects, one prominent drug in HIV treatment, efavirenz (EFV), possesses similar psychoactive properties to the hallucinogenic drug lysergic acid diethylamide (LSD), leading to symptoms such as mania and psychosis [62,107]. Additionally, both EFV and ritonavir (RTV) produce euphoric effects when combined with substances such as methamphetamine, ecstasy, heroin, tobacco, and cannabis [53,108,109]. ARVs may potentially be diverted for “alternative” uses, such as the “breast enhancement” recorded in Nigeria with Combivir [lamivudine (3TC) and zidovudine (ZDV)] [110]. Another study by Inciardi et al. reported the recreational use of EFV and RTV in clubs and street populations [111]. In South Africa, the recreational use of ARTs is referred to as whoonga or nyaope. Certain nyaope mixtures have also been reported to contain HIV ARVs such as EFV, RTV, and nevirapine (NVP) [17,38,45,49,50,51,54,60,76] (Table 3). As a result of the misuse of ARVs, the development of resistance to ARVs can occur. Therefore, with the rise of drug abuse in South Africa and other countries, there is limited data on drug–drug interactions and their impact on ART adherence. In these situations, prospective patients frequently disregard the recommended course of treatment. 

A recent systemic review by Varshney et al. investigated the risks and consequences associated with the usage of nyaope and the recreational use of ARVs in South Africa [112]. Findings from this review documented that, due to the inclusion of ARVs in nyaope, poor adherence to ARV treatment occurs in HIV-infected individuals who are nyaope users, and that users are often at risk for hepatitis B and C, tuberculosis, and infective endocarditis infections [43,46,64]. HIV-positive nyaope users were more likely to engage in high-risk behaviours and to have high viral loads [46]. Furthermore, HIV-positive nyaope users were involved in transactional sex to finance their nyaope use [60], injected drug cocktails [60,64], and had poor adherence to ARV treatments [46]. Therefore, the inclusion of ARVs in some nyaope mixtures could lead to the emergence of drug resistance and ultimately lead to poor response to HIV treatment. This may potentially lead to an increase in the development of drug-resistant strains in those who use these drugs outside of the prescribed instructions, and may also potentiate pre-treatment drug resistance in those that have not started treatment, especially those that do not know their HIV status. The complexity of ARV abuse highlights the need for additional investigation and treatment interventions to address the potential hazards and negative effects associated with the non-medical use of these medications. Table 3 shows a summary of studies that have investigated the recreational use of ARVs worldwide.

## 10. Impact of Substance Abuse in HIV-Infected Populations

Alcohol and substance use are commonly believed to impact HIV viral control by affecting the nonadherence to ARV medications [113,114,115,116,117]. As ARTs have become widely available, active alcohol and substance use negatively affect the goal of achieving and maintaining an undetectable HIV plasma viral load. According to some studies, substance use (methamphetamine, cocaine, opioids, and cannabis) may directly impact immune activation pathways and facilitate HIV replication [118,119,120,121,122]. In addition, drug abuse is associated with increased HIV infection, neurocognitive impairment, HIV replication, morbidity, and mortality when compared to HIV-uninfected drug users and HIV-infected non-drug users [123,124]. Cannabis, cocaine, morphine, methamphetamine, heroin, and amphetamine are the most commonly abused illicit drugs in HIV-infected populations worldwide. 

HIV-infected individuals receive ART to suppress viral replication and control disease progression. However, with the increasing incidence of SUD among HIV-infected individuals, several factors, such as the decreased adherence to ART treatment, uncontrolled viraemia, and increased chronic inflammatory disease, are possible causes of reduced CD4+ T-cell reconstitution in this population group [125,126]. The use of drugs such as methamphetamine also increases the risk of HIV infection worldwide [127,128]. Among other addictive drugs, methamphetamine has been revealed as a highly addictive stimulant that increases sexual desire, self-confidence, and which provides sustained energy as well as decreasing inhibitions [129]. As a result of these physiological effects, methamphetamine users may be more likely to engage in high-risk behaviours of HIV transmission, such as having multiple partners, not using condoms, and trading sex for money or drugs [130]. Evidence from other South African studies about risky behaviours associated with methamphetamine use exists even among users of other substances [131,132]. Adherence to ARTs can be negatively impacted by substance addiction, especially alcohol and drug dependence. Drug users who are HIV-infected can find it difficult to stick to their prescribed medications, which could result in inadequate viral suppression and a higher chance of disease progression.

## 11. Impact of Drug Abuse on Drug Metabolism

It has also been established that substance abuse, such as of alcohol, tobacco, and methamphetamine, decrease the adherence to ART treatment in HIV-infected individuals [133]. Therefore, drugs of abuse may potentially interfere with the ART drug metabolism in HIV-infected individuals. A review by Kumar et al. proposed that common metabolic enzymes involved in the metabolism of ART and alcohol (CYP2E1 and CYP3A4), nicotine (CYP1A1, CYP2A6, and CYP3A4), methamphetamine (CYP2D6 and CYP3A4), cocaine/opioids (CYP3A4), and marijuana/tetrahydrocannabinol (CYP2C9) may result in an altered bioavailability of ART drugs (the ability of a drug or other substance to be absorbed and used by the body) [134]. Subsequently, drug–drug interactions between drugs of abuse and ART drugs mediated by CYP enzymes may result in the decreased adherence to ART, leading to an increased HIV replication in HIV-infected individuals [134]. Although highly active ART has been a great success, new challenges have arisen that could lead to the failure of the treatment [135,136]. One of these challenges is the appearance of interactions between ART drugs and medications or illicit drugs taken concurrently [135,136]. Consequently, as some nyaope cocktails contain ARVs, nyaope use could also lead to the poor adherence to ART drugs. Currently, no studies have evaluated how nyaope mixtures—especially those containing antiretrovirals—affect virus replication, drug interactions, and/or cellular cytotoxicity. Therefore, it is crucial to study the effects of nyaope and drug interactions on the viral replication of the BBVs that are prevalent in nyaope users.

## 12. Alcohol and HIV Infection

Studies have been conducted on the impact of alcohol and viral infections. HIV infection and transmission can be worsened by alcohol, reducing viral suppression, increasing the emergence of viral resistance to ARV therapies, and opportunistic infections and progression that can be exacerbated by alcohol-associated immunosuppression [137,138]. In vitro, HIV gp120 and alcohol increase the permeability of the blood–brain barrier [139]. Other studies have found that alcohol increased HIV replication in cells such as T lymphocytes, monocyte-derived macrophages, monocyte-derived dendritic cells, epithelial cells, and oral keratinocytes [116,140]. Based on the 2008 population-based survey report in South Africa, hazardous, harmful, and dependent alcohol use was 9.0% among women, 17.0% among men, and 2.9% among individuals 15 years and older [141]. In 2015, alcohol was reported to be the fifth leading cause of death and disability [142], which is, therefore, the leading cause of sexually transmitted infections, unemployment, criminal activities, and interpersonal violence in South Africa [143,144]. A study conducted in Tshwane Metropole in South Africa established that 53% of individuals heavily consume alcohol (70% men and 30% women) [145]. The Tshwane Metropole, as well as other areas in South Africa, has also been reported to have a high prevalence of substance abuse by various media reports and research studies [69,146,147].

## 13. Drugs of Abuse and Viral Hepatitis (HBV and HCV)

PWIDs are at risk of contracting HBV and HCV infection. PWIDs frequently share needles, which is the main route of transmission of HBV and HCV [148,149]. Although HBV is vaccine-preventable, it remains the main leading cause of death worldwide. According to a global systematic review of HBV infection conducted across 161 countries, HBsAg prevalence was estimated at 4%, with the highest endemicity in Africa (a total of 9%) and the Western Pacific (a total of 5%) [150]. In observational studies of the general population, healthcare workers, and pregnant women, the seroprevalence of hepatitis B surface antigen (HBsAg) was estimated to be 7%, indicating a high intermediate endemicity, with an estimated 3.5 million chronically infected individuals, and ~70% are infected with the HBV subgenotype A1 in South Africa [151]. In South Africa, the seroprevalence of HCV and identifiable risk factors are still poorly understood and are being characterised. The prevalence of HCV is estimated to be <1%, which indicates that 600,000 people are infected with the virus based on HCV antibody (anti-HCV) status [152]. South Africa has limited data on HBV and HCV infections in PWIDs, with the latest data from a study that was performed in three South African cities. According to Scheibe et al., HBV and HCV prevalence rates in South African PWIDs are 5% and 55%, respectively [10]. Infection with chronic HBV and HCV causes significant liver-related morbidity and mortality worldwide. However, HCV can be cured by new direct-acting antiviral (DAA) therapies, leading to a sustained viral response. An HBV cure remains a challenge due to persistent covalently closed circular DNA (cccDNA) in the liver, which is the major barrier to eliminating chronic hepatitis B infection [153]. According to a global systemic review conducted by Degenhardt and colleagues, PWUDs are more likely to have a high HCV burden than other people, with over 50% prevalence estimates among them [8]. Opioids and other drugs of abuse have also been found to increase HBV replication in vitro. Evidence shows that opioid receptors are expressed in the liver, where they are important mediators of liver disease [154,155,156]. Furthermore, it has been established that multiple opioid receptors are expressed by hepatic stellate cells (HSCs), which ultimately contribute to liver fibrosis [154,155,156]. In addition, morphine, heroin, and methamphetamine have been shown to increase HCV replication in vitro [157,158,159]. A recent report by Dr. Blackard’s group from the USA demonstrated that fentanyl increases HCV and HBV replication in Huh7.5_JFH1_ and HepG2.2.15 hepatocyte cell lines and leads to significant apoptosis of hepatocyte cell lines [88]. Therefore, the abuse of drugs and other substances may ultimately have an impact on viral hepatitis and liver disease progression.

## 14. Alcohol and Viral Hepatitis (HBV and HCV)

Globally, HBV, HCV, as well as excessive alcohol use causes liver injury. Since the liver is a primary site for both HBV and HCV replication and ethanol metabolism, the ethanol metabolism is often associated with viral hepatitis. An alcohol-related liver disease accompanied by hepatitis virus accelerates the disease’s progression to hepatocellular carcinoma (HCC) [160]. Existing evidence from the literature reported that alcohol consumption contributes to apoptosis, oxidative stress, immune dysfunction (lower cytotoxic T-cell and helper T-cell activity and decreased cytokine production), increased HCV viral load, and increased HCV quasispecies; however, the mechanisms by which alcohol alters HCV replication and disease are largely unknown [161,162,163]. Studies have reported that a synergistic hepatotoxic effect of alcohol and HCV infection increased the risk of advanced liver disease, the rapid progression of fibrosis, and a higher prevalence of liver cancer (HCC) [164,165]. In vitro studies have also reported an increased viral replication of HCV following the exposure of permissive cells to alcohol [166]. Additionally, individuals who are HBV-infected are at greater risk of fibrosis as a result of alcohol abuse [167], as well as enhancing liver necroinflammation changes in HBsAg-positive patients [168]. A study by Larkin et al. described alcohol consumption to increase HBsAg and viral DNA in transgenic mice [169]. In addition, another study demonstrated increased HBsAg levels in HepG2 cells in the presence of ethanol [170]. Nevertheless, the mechanisms by which alcohol impacts HBV replication and disease are not well studied.

## 15. Recommendations

Even though nyaope is becoming more prevalent through smoking and injections in South Africa, the South African government has not fully embraced the harm reduction that accompanies nyaope use [60]. South Africa has a limited number of harm-reduction programmes, with only the City of Tshwane Municipality sponsoring the Community-Oriented Substance Use Programme (COSUP), which is South Africa’s first publicly funded harm-reduction intervention for unregulated drugs [72,146]. The Ivan Toms Centre for Men’s Health (ITCMH), operating under the Anova Health Institute in Cape Town, provides MSM primary healthcare services on sexual health [11]. These services include HIV testing, antiretroviral therapy, STI diagnosis and treatment, and post-exposure prophylaxis (PEP) and pre-exposure prophylaxis (PrEP) [11]. In 2012, the institution established an MSM-targeted drug harm-reduction programme, including a needle exchange and without opioid-substitution therapy, funded by the AIDS Fonds, Mainline, the Netherlands, to offer harm-reduction services to MSM who are drug users [11]. Yet, during this period, it was established that 27% of the MSM participants tested positive for antibodies to HCV. This shows that BBV infections continue to predominate in MSM who are also IDUs. Therefore, future research should evaluate the effectiveness of alternative therapies and antiviral medications for treating BBVs among IDUs. As a result, treatment approaches could be identified and treatment outcomes could be improved.

Without accessible harm-reduction programmes, PWIDs are at high risk for BBVs (HIV, HBV, and HCV). The coverage of needle, syringe, and opioid substitution therapy (OST) services in South Africa is below the global recommendations, and no hepatitis services currently exist for PWIDs [10], which requires strong intervention for harm reduction in drug users. South Africa is considered to be one of the most endemic countries worldwide, with high rates of SUD, despite the limited data available on BBVs among drug users. Nyaope is a commonly abused drug in many parts, if not all areas, in South Africa, and appears to be the most abused drug among the youth. It continues to cause detrimental effects on the health and well-being of individuals that abuse it. As a result, it is not surprising that injecting nyaope increases the risk of the transmission of BBVs, as with other abused drugs. In addition, nyaope is thought to be exceptionally potent when compared to other substances, with serious health effects and addiction. A worrying component of nyaope mixtures is the presence of ARVs, which are drugs used for the treatment of HIV infection. It is believed that this practice stems from the belief that ARVs can enhance the effects of nyaope. However, the misuse of ARVs in this manner poses significant risks to individuals, as these medications are designed for specific medical purposes and should only be taken under professional guidance. In addition to ARVs, nyaope often contains other substances commonly associated with drug abuse, such as cannabis and heroin. The inclusion of rat poison and detergent further highlights the hazardous nature of this drug. These substances are not intended for human consumption and can have severe toxic effects on the body. It is worth noting that the chemical composition of nyaope is not consistent and can vary over time. The variability of these components adds to the unpredictability and dangers associated with nyaope, making it even more challenging to address from a health and law enforcement perspective. 

Given how potent nyaope is and how its chemical composition is constantly changing, it is crucial to increase public awareness about its risks and implement effective strategies to combat its production, distribution, and usage. To address the complexities and dangers associated with nyaope and to protect individuals from its detrimental effects, significant effort needs to be made to identify effective prevention and treatment strategies that address the complex social and environmental factors that contribute to viral infections among drug users, such as public health measures, law enforcement actions, and educational campaigns. Hence, more studies of drugs of abuse like nyaope and viral diseases are warranted. Studies, such as in vitro studies, are also essential to evaluate the impact of nyaope on HIV, HBV, and HCV replication and biomarkers of disease progression. In addition, cohort studies of nyaope users to understand the overall burden of HIV, HBV, and HCV, as well as the impact of nyaope use on immune dysfunction and viral levels, as well as the biomarkers of disease progression, are needed. Previous studies have demonstrated that opioids increase HIV and/or HCV replication in vitro and in vivo [88,89,171]. Moreover, higher viral loads have also been associated with increased transmission as well as accelerated disease progression [46,125]. Therefore, more research is required to determine effective ways of preventing viral infections in PWID/UDs. This may involve developing new interventions or assessing existing programs to identify effective ones, such as harm-reduction programmes and OST services, as well as developing vaccination and treatment programs for hepatitis B and C infection.

## 16. Conclusions

The use of nyaope is on the rise in South Africa, especially among young people, and it has become a growing public health concern. Nyaope continues to cause detrimental effects on the health and well-being of individuals who abuse it. As a result, it is not surprising that injecting nyaope increases the risk of transmission of BBVs, as with other abused drugs. With evidence that opioid receptors are expressed in multiple cell types that are relevant to HIV and/or HBV/HCV pathogenesis, we expect nyaope to interact with opioid receptors as well as cannabinoid receptors. Therefore, it is crucial to examine the interaction between drugs of abuse, such as nyaope, and viral infections, as well as their impact on viral pathogenesis in vitro and in vivo for the development of effective treatment strategies specifically for PWID/UDs.

## Figures and Tables

**Table 1:** Summary of studies conducted on drugs of abuse and associated blood-borne viruses.

Ref	Details of the Study		Types of Drugs Investigated	BBVs	Findings
[9]	Country	South Africa	Heroin and ATSs	HIV only	A total of 14% were HIV-positive (18% female and 13% male), 22% had STI symptoms in the past year, and 51% were female sex workers.
	Study design	Cross-sectional study			
	Study population	PWIDs			
	Sample size	450			
[10]	Country	South Africa	Heroin, methamphetamine, or ATSs	HIV, HBV, and HCV	A total of 21% were HIV-positive, 5% were HBV-positive, and 55% were HCV-positive. A total of 52% used condoms during their last penile–vaginal sexual encounter, and 6% traded commodities or drugs for sex.
	Study design	Cross-sectional study			
	Study population	PWIDs			
	Sample size	943			
[11]	Country	South Africa	Heroin and crystal methamphetamine	HIV, HBV, and HCV	A total of 27% were HCV-positive, 2% were HBV-positive, and 38% were HIV-positive.
	Study design	Cross-sectional study			
	Study population	MSM participants who were injecting drug users			
	Sample size	41			
[13]	Country	United States of America	Opioids, ATSs, and cocaine	HIV and HCV	Injection drug users reported higher rates of HCV infection (53% vs. 3%) and HIV infection (6% vs. 2%) compared to the general population.
	Study design	Cross-sectional study			
	Study population	Patients with opioid use disorder and those who use injecting drugs			
	Sample size	2200			
[14]	Country	United States of America	Methamphetamine, cocaine, and heroin	HIV, HBV, and HCV	A total of 11% of participants were HIV-positive, 78% were HBV-positive, and 71% were HCV-positive. A total of 51% were positive for BBVs, and 6% were aged 50–59 years. A total of 26% lived below the poverty line, with 49% using methamphetamine.
	Study design	Cross-sectional study			
	Study population	People who reported they had used methamphetamine at some point in their lives			
	Sample size	50,588			
[12]	Country	United States of America	Heroin, methamphetamine, cocaine, crack cocaine, prescription opioids, tranquilizers, stimulants, and sedatives, and speedball (heroin/cocaine) and goofballs (heroin/methamphetamine)	ND	In the last six months, the most common negative outcomes of injections were blood on clothes or surfaces (40%), the recipient’s blood on the injection provider (23%), and missing a vein (17%).
	Study design	Cross-sectional study			
	Study population	Participants who reported having injected drugs in the last 6 months			
	Sample size	336			
[15]	Country	Ghana	Marijuana, crack, cocaine, heroin, crystal meth/methamphetamine, tramadol, and pethidine	HIV, HBV, and HCV	A total of 3% were HIV-positive, 4% were HBV-infected, and 7% for HCV among drug users.
	Study design	Cross-sectional study			
	Study population	PWIDs			
	Sample size	323			
[8]	Country	Global systemic review on the prevalence of BBVs among IDUs	ND	HIV, HBV, and HCV	Globally, 17% of PWIDs have HIV, 52% have HCV antibodies, and 9% have HBV surface antigens. A total of 83% inject opioids, 33% inject stimulants, 28% of PWIDs are younger than 25 years, 22% are homeless, and 58% have been incarcerated in the past.
	Study design	Systematic review that systematically reviewed grey and peer-reviewed literature through a multistage process			
	Study population	PWIDs			
	Sample size	55,671 papers and reports			

Ref = reference; ND = not done; IDUs = injecting drug users; BBVs = blood-borne viruses; ATSs = amphetamine-type stimulants; STIs = sexually transmitted infections; PWIDs = people who inject drugs; HIV = human immunodeficiency virus; HCV = hepatitis C virus; HBV = hepatitis B virus; STI = sexually transmitted infection; OST = opioid substitute therapy; NHANES = National Health and Nutrition Examination Survey; MSM = men who have sex with men; ED = emergency department; PRISMA = Preferred Reporting Items for Systematic Reviews and Meta-Analyses; GATHER = Guidelines for Accurate and Transparent Health Estimates Reporting.

**Table 2:** Summary of nyaope studies conducted in South Africa.

Ref	Details of the Study		Names Used for the Drug	BBVs	Nyaope Composition	Findings/Risk Factors Associated with Nyaope Use
[72]	Province and/or location	Gauteng, Tshwane	Nyaope	ND	ND	Nyoape problem in Tshwane is attributed to the following three community issues: inadequate social services, school facilitators, and strained police–community interactions. These factors, along with unfavourable home environments and the lack of religious motivation, contribute to nyoape usage.
	Study design	Exploratory descriptive qualitative study				
	Methodology	Focus group interviews				
	Total nyaope users/total participants	19				
[54]	Province and/or location	Western Cape, Cape Town metropolitan area	Nyaope, Whoonga	HIV	EFV	More research is needed to classify recreational ART usage as a substance of abuse and to prevent HIV and substance abuse. Best practices for prescribing ART are needed to reduce diversion and mitigate medication resistance. Increased recreational use could increase HIV infection and blood-borne illnesses.
	Study design	Cross-sectional study				
	Methodology	Focus group discussions and questionnaire interviews				
	Total nyaope users/total participants	200				
[56]	Province and/or location	Gauteng, Tshwane	Nyaope	ND	ND	The external locus of control consists of the addictive nature of the drugs, the influence of friends and associates, drug traffickers, and the police’s inability to apprehend dealers.
	Study design	Qualitative and explorative study				
	Methodology	In-depth interviews and focus group discussions				
	Total nyaope users/total participants	123				
[36]	Province and/or location	Gauteng	Nyaope	ND	ND	Nyaope addiction worsens South Africa’s mental health crisis, necessitating a strategy for community-based rehabilitation, considering the vast number of users compared to available resources.
	Study design	NS				
	Methodology	NS				
	Total nyaope users/total participants	NS				
[44]	Province and/or location	Gauteng, rehabilitation centres, and urban areas across Pretoria	Nyaope	ND	ND	A total of 52% of nyaope addicts are in recovery centres, with 72% attempting to quit alone. A total of 90% lack a formal income, and treatment costs make rehabilitation unviable for many, despite their desire to quit.
	Study design	Cross-sectional study				
	Methodology	Questionnaires				
	Total nyaope users/total participants	221				
[57]	Province and/or location	Gauteng, rehabilitation facility in the West Rand Municipality	Nyaope	HIV	ND	At baseline, female participants had a higher prevalence of mental illness, sexual trauma, and HIV infection than males, with 25% using heroin, 40% being HIV-positive, and 50% being sex workers.
	Study design	Prospective study				
	Methodology	Questionnaires				
	Total nyaope users/total participants	300				
[17]	Province and/or location	Gauteng	Nyaope	ND	Nicotine, α-caryophyllene, α-humulene, α/β-selinene, phenacetin, neophyltadiene, caffeine, palmitic acid, phytol, cannabivarol, tetrahydrocannabivarin, cannabidiol, NVP, cannabichromene, Δ9-tetrahydrocannabinol, cannabigerol, acetylcodeine, cannabinol, 6-monoacetylmorphine, diamorphine	Optimal organic solvent was found to maintain the stability of the ARVs EFV and NVP as well as common nyaope components during extraction and autosampler analysis.
	Study design	NS				
	Methodology	The study determined the most suitable organic solvent in which the common components of nyaope are stable during extraction and prior to the analysis of nyaope samples using GC-MS				
	Total nyaope users/total participants	NS				
[77]	Province and/or location	Gauteng	Nyaope	ND	Morphine, heroin, codeine, and caffeine	Nyaope, a drug equivalent to heroin, increased LDH levels in SH-SY5Y cells, reduced Bax and Bcl-2 mRNA expression, and elevated LC3 mRNA expression, potentially activating necrosis and autophagy pathways.
	Study design	NS				
	Methodology	Chemical analysis of nyaope samples was performed using the GC/MS and in vitro studies were performed using the SH-SY5Y neuroblastoma cells to determine the cytotoxic effect of nyaope post-drug exposure using different drug concentrations				
	Total nyaope users/total participants	NS				
[53]	Province and/or location	Gauteng	Plazana, BoMkon, Unga	HIV	EFV	With a complex and polymodal receptor pharmacology and animal model studies primarily demonstrating serotonergic action, EFV is the primary cause of NPAEs and illegal ARV use.
	Study design	NS				
	Methodology	NS				
	Total nyaope users/total participants	NS				
[52]	Province and/or location	Eastern Cape, Buffalo Flats high schools	Nyaope, Kataza, Whoonga	ND	Marijuana, “tik” (methamphetamine), cocaine, mandrax, ecstasy, heroin	Learners frequently use alcohol, tobacco, and marijuana on school grounds, with restrooms and athletic fields being the primary locations.
	Study design	Cross-sectional descriptive study				
	Methodology	Self-administrated questionnaire				
	Total nyaope users/total participants	242				
[40]	Province and/or location	Gauteng, Johannesburg	Kataza	ND	Marijuana, methamphetamine	A single mother’s family structure, poor care, domestic problems, and teacher support were all factors in the common misuse of substances by students, including alcohol, cigarettes, marijuana, and dagga.
	Study design	Qualitative and explorative study				
	Methodology	Focus group interview				
	Total nyaope users/total participants	5				
[49]	Province and/or location	KwaZulu-Natal	Whoonga	ND	EFV, rat poison, household cleaning detergent (Handy Andy)	Whoonga, an addictive drug cocktail containing ARVs like EFV, is believed to be dangerous for health and to increase HIV infection risk in individuals as young as 14 years.
	Study design	Qualitative and explorative study of sexual activities				
	Methodology	Semi-structured interviews				
	Total nyaope users/total participants	13				
[38]	Province and/or location	Gauteng, Soweto	Whoonga	HIV	EFV	Abuse of ARVs could have adverse effects on the health and safety of HIV-positive patients, as well as healthy individuals who abuse ARVs.
	Study design	Qualitative and explorative study				
	Methodology	Semi-structured interviews				
	Total nyaope users/total participants	43				
[39]	Province and/or location	Gauteng, Tshwane	Whoonga, Nyaope	HIV	EFV	The majority of ARV users and their families are aware of the harmful effects of the drug, especially its hallucinogenic qualities, which are abused by street gangs as “nyaope”, increasing the prevalence of HIV and AIDS.
	Study design	Qualitative and descriptive study				
	Methodology	Semi-structured interviews				
	Total nyaope users/total participants	26				
[41]	Province and/or location	Mpumalanga, Umjindi	BoMkon, Nyaope, Whoonga	ND	Heroin, dagga, rat poison, ARVs	The drug is a contributing factor to increased crime and domestic violence.
	Study design	NS				
	Methodology	NS				
	Total nyaope users/total participants	NS				
[35]	Province and/or location	Limpopo, Mpumalanga, KwaZulu-Natal	Nyaope, Sugars, Pinch	ND	ND	Many users of nyaope regret their initial experimentation, often due to physical aches, frequent consumption, and a reputation for stealing to support their habit.
	Study design	Qualitative study				
	Methodology	In-depth interviews and focus group discussions				
	Total nyaope users/total participants	94				
[62]	Province and/or location	Western Cape, Cape Town	Nyaope, Whoonga, Wunga	HIV	ND	Adolescent drug and alcohol use increases whoonga use, with 3% using off-label ARTs for recreational purposes.
	Study design	Cross-sectional study				
	Methodology	Surveys				
	Total nyaope users/total participants	6				
[47]	Province and/or location	Eastern Cape, mission location of Butterworth	Nyaope	ND	ND	Nyaope use poses health risks, including hallucinations and delusions in young people. Addiction can cause mood swings, conflicts, and negatively impact future prospects, making it a crucial drug for adolescents.
	Study design	Explorative and descriptive qualitative study				
	Methodology	In-depth interviews				
	Total nyaope users/total participants	7				
[34]	Province and/or location	Gauteng, Mpumalanga, North West Province	Nyaope	ND	ND	Users, their families, and the community are expressing a cry for help in breaking free from their addiction.
	Study design	Explorative qualitative study				
	Methodology	Focus group discussions, in-depth interviews, and participant-administered questionnaires				
	Total nyaope users/total participants	108				
[73]	Province and/or location	Gauteng, City of Tshwane Municipality	Nyaope, Whoonga	HIV	ND	Women injecting nyaope are at a higher risk of contracting HIV and often engage in transactional sex to finance their use.
	Study design	Qualitative study				
	Methodology	Semi-structured interviews				
	Total nyaope users/total participants	24				
[43]	Province and/or location	Gauteng, and streets from the urban areas of Ga-Rankuwa, Soshanguve, and Hammanskraal in Pretoria	Nyaope	ND	ND	Nyaope, an addictive drug, provides euphoria, confidence, and stress relief, but can cause irreparable harm due to its widespread availability and lack of law enforcement.
	Study design	Explorative qualitative				
	Methodology	Semi-structured interviews				
	Total nyaope users/total participants	68				
[67]	Province and/or location	Gauteng, Hammanskraal in Pretoria	Nyaope	ND	ND	The government’s efforts to combat substance abuse are not adequately serving the community, as drug availability and unemployment can lead to drug misuse. Prioritising vocational training and employment prospects can help support recovering addicts.
	Study design	Qualitative study				
	Methodology	Semi-structured interviews				
	Total nyaope users/total participants	6				
[71]	Province and/or location	KwaZulu-Natal, Durban	Nyaope, Whoonga	ND	ND	Most consumers are addicted to smoking, with initiation narratives excluding injectable drug use. Participants noted racial differences between heroin users who smoke and those who inject the drug.
	Study design	Qualitative study				
	Methodology	Semi-structured interviews				
	Total nyaope users/total participants	30				
[69]	Province and/or location	86 specialist treatment centres across South Africa, with 20 in Gauteng, 14 in KZN, 29 in Western Cape, 9 in Eastern Cape, 9 in Limpopo and Mpumalanga, and 6 in Free State, Northern Cape, and North West	Nyaope	HIV	ND	The percentage of injectable drug use in patients increased from 2% in 2013 to 4% in 2017, with nyaope users having lower HIV test likelihoods. Annual nyaope-related admissions ranged from 145 to 1000 (with the lowest in 2012 and the highest in 2015).
	Study design	Cross-sectional study				
	Methodology	Questionnaires				
	Total nyaope users/total participants	NS				
[70]	Province and/or location	Gauteng, Mabopane, Ga-Rankuwa, and Ga-Rankuwa View in Pretoria	Nyaope, Whoonga, Pinch, Unga	ND	ND	A total of 92% of participants experienced erectile dysfunction, with nyaope negatively impacting sexual function, especially in individuals with hyperprolactinaemia and hypogonadism.
	Study design	Cross-sectional study				
	Methodology	Researcher-developed demographic questionnaire, International Index of Erectile Function Questionnaire, blood samples				
	Total nyaope users/total participants	50				
[61]	Province and/or location	Gauteng, substance treatment centre in Tshwane	Nyaope	ND	ND	Drug use is primarily driven by peer pressure (55%), stress (27%), and family issues (16%), with marijuana (29%) and nyaope (66%) being the most commonly used drugs.
	Study design	Cross-sectional, quantitative descriptive study				
	Methodology	Self-administered questionnaire, quantitative and descriptive survey				
	Total nyaope users/total participants	141				
[68]	Province and/or location	KwaZulu-Natal, peri-urban township in Durban	Nyaope, Whonoga, Sugars, Kataza	ND	ND	Whoonga exploration can quickly escalate into addiction, often leading to criminal activities to finance high drug-use demands.
	Study design	Case report				
	Methodology	Interpretative phenomenological approach, with direct accounts from participants and family members				
	Total nyaope users/total participants	1				
[59]	Province and/or location	KwaZulu-Natal, Inkosi Albert Luthuli Central Hospital in Durban	Nyaope, Whoonga	ND	ND	Nyaope use during pregnancy led to infants experiencing various symptoms, including malnutrition, dysmorphia, growth restriction, and respiratory distress, leading to one infant’s death.
	Study design	Case report				
	Methodology	Patient records review				
	Total nyaope users/total participants	2				
[65]	Province and/or location	Gauteng, Chris Hani Baragwanath Academic Hospital in Soweto	Nyaope	ND	ND	Two mothers used nyaope during pregnancy, with one treating withdrawal symptoms with methadone and clonazepam. Infants with nyaope mothers experienced retinopathy, seizures, growth restriction, and hypoglycaemia.
	Study design	Case report				
	Methodology	Patient records review				
	Total nyaope users/total participants	2				
[64]	Province and/or location	Gauteng, Chris Hani Baragwanath Academic Hospital in Soweto	Nyaope, Whoonga, Wunga	HIV	ND	Three males, all HIV-positive, were injecting nyaope users and presented with septic pulmonary emboli and tricuspid regurgitation, indicating nonadherence to treatment.
	Study design	Case report				
	Methodology	Patient records review				
	Total nyaope users/total participants	3				
[46]	Province and/or location	Gauteng, Chris Hani Baragwanath Academic Hospital in Soweto	Nyaope, Whoonga, Wunga	HIV, HBV, and HCV	ND	The population frequently experienced HIV-related immunosuppression and co-infection with hepatitis C, with nyaope usage leading to frequent clinical symptoms such as dyspnea (87%), fever (59%), right ventricular failure (43%), withdrawal symptoms (25%), and peripheral suppurative infection (9%). A total of 8% were HBV-positive and 58% were HCV-positive.
	Study design	Retrospective cohort study				
	Methodology	Patient files from hospital records				
	Total nyaope users/total participants	68				
[66]	Province and/or location	Gauteng, Weskoppies Hospital Substance Rehabilitation Unit	Nyaope	ND	ND	Nyaope use is linked to drug addiction treatment failure, with psychotic illnesses, substance-induced disorders, and Cluster B personality characteristics prevalent, with tobacco, alcohol, and cannabis being the most commonly used substances.
	Study design	Retrospective cohort study				
	Methodology	Clinical files, Substance SRU referral forms, SRU attendance, computerised demographic records, nursing notes, and administration files from the hospital				
	Total nyaope users/total participants	18				
[63]	Province and/or location	Gauteng and Western Cape, HIV Voluntary Testing and Counselling Centres in Johannesburg and Cape Town	Nyaope, Whoonga	HIV	ND	The HIV positivity rate was 67%, and recreational ARV use was not significantly linked to viral suppression at nine months, with most participants using ARTs for the first time.
	Study design	Prospective cohort study				
	Methodology	Surveys and assessment of viral load				
	Total nyaope users/total participants	24				
[58]	Province and/or location	Gauteng, Soweto	Nyaope			Nyaope users experience cortical alterations in impulse control, decision making, executive processes, and social perception, with significant grey matter loss/atrophy.
	Study design	Case-control study				
	Methodology	Written informed consent (MRI scans of nyaope-using males and controls, along with analyses of the scans)				
	Total nyaope users/total participants	28				

Some studies or reports reported HIV infection but did not report the HIV prevalence rate, and, in some cases, no other sexually transmitted infections (STIs) were reported. Ref = reference; NS = not specified; ND = not done; COSUP = Community-Oriented Substance Use Programme; OST = opioid substitution therapy; SRU = substance rehabilitation unit; GC-MS = gas chromatography–mass spectrometry; NPAEs = neuropsychiatric adverse events; EFV = efavirenz; NVP = nevirapine; KZN = KwaZulu-Natal; SANCA = South African National Council on Alcoholism and Drug Dependence; SACENDU = South African Community Epidemiology Network on Drug Use; CoPs = communities of practice; HIV = human immunodeficiency virus; AIDS = acquired immune deficiency syndrome; HCV = hepatitis C virus; HBV = hepatitis B virus; ARVs = antiretrovirals; ART = antiretroviral therapy.

**Table 3:** Summary of studies that investigated the recreational use of ARVs.

Ref	Details of the Study		Drug Compositions	Clinical Outcome	Findings
[50]	Country	South Africa	NS	The misuse of ARVs in HIV-infected individuals and those who abuse them may lead to the development of drug-resistant strains and potential side-effects.	HIV-infected individuals are reportedly smoking ARVs instead of using them as prescribed, with patients and healthcare staff selling the drugs for money.
	Study design	NS			
	Total nyaope users/total participants	NS			
[76]	Country	South Africa	EFV	NS	Misuse of ARVs has the potential to create HIV strains that are resistant to EFV.
	Study design	Exploratory study			
	Total nyaope users/total participants	Over 120 patients			
[51]	Country	South Africa	EFV	NS	The high prevalence of HIV in southern Africa necessitates effective treatment strategies to address emerging issues like recreational ARV use.
	Study design	Comment/report on recreational use of ARVs in South Africa			
	Total nyaope users/total participants	ND			
[49]	Country	South Africa	EFV	NS	Additional substances including rat poison and Handy Andy, a household cleaning detergent, have been implicated in the composition of the nyaope drug.
	Study design	Qualitative and explorative study			
	Total nyaope users/total participants	13			
[38]	Country	South Africa	EFV	NS	Concerns about drug theft and safety risks were expressed by participants who reported smoking, whoonga, and crushed ARVs—possibly EFV—combined with illicit substances.
	Study design	Qualitative and explorative study			
	Total nyaope users/total participants	43			
[33]	Country	South Africa	ZDV	ND	Samples consistently detected caffeine, drugs of abuse, antibiotics, antiretrovirals, CNS depressants, and stimulants, with some containing citrofex and ZDV, and some containing antibiotics and ARVs.
	Study design	Cross-sectional, qualitative, and descriptive pilot study			
	Total nyaope users/total participants	40 nyaope samples from 12 areas in Gauteng province			
[54]	Country	South Africa	EFV	A total of 42% said they were living with HIV.	Adolescents use non-prescribed ARTs for recreational purposes, with 71% using smoking, 15% using snorting, 15% using injecting, 15% using ingesting, and 3% using inserting methods.
	Study design	Descriptive quantitative study			
	Total nyaope users/total participants	200			
[110]	Country	Nigeria	Combivir (3TC and ZDV)	The patient developed resistance to NRTIs after a blood transfusion, despite having full sensitivity to NNRTIs and PIs, and despite weeks of unsuccessful HIV treatment.	The findings highlight the crucial issues to be addressed concerning the misuse of ARVs and the consequences of the recreational use of ARVs.
	Study design	Case report			
	Total nyaope users/total participants	1			
[111]	Country	United States of America	RTV, EFV	A total of 29% of participants used prescription drugs, with polydrug usage being prevalent. Drug addicts from the streets and clubs have reported receiving their prescription medicines from HIV-positive friends. Most of the participants had trouble adhering to their ARV treatment regimens.	Doctors prescribing potentially abused medications should be vigilant, especially for elderly and HIV-positive patients who were previously at a low risk for drug abuse.
	Study design	Explorative qualitative study			
	Total nyaope users/total participants	74			

Some studies or reports reported HIV infection but did not report the HIV prevalence rate, and, in some cases, no other sexually transmitted infections (STIs) were reported. Ref = reference; NS = not specified; ND = not done; EFV = efavirenz; KZN = KwaZulu-Natal; HIV = human immunodeficiency virus; HCV = hepatitis C virus; HBV = hepatitis B virus; ARVs = antiretrovirals; ART = antiretroviral therapy; EFV = efavirenz; ZDV = zidovudine; 3TC = lamivudine; RTV = ritonavir; GC-MS = gas chromatography–mass spectrometry; TOF-MS = time-of-flight mass spectrometry; NRTIs = nucleoside reverse transcriptase inhibitors; NNRTIs = non-nucleoside reverse transcriptase inhibitors; PIs = protease inhibitors; CNS = central nervous system; MDA = methylenedioxyamphetamine.
